# Spatial and temporal variation in sponge spicule patches at Station M, northeast Pacific

**DOI:** 10.1007/s00227-014-2609-1

**Published:** 2015-01-22

**Authors:** C. Laguionie-Marchais, L. A. Kuhnz, C. L. Huffard, H. A. Ruhl, K. L. Smith

**Affiliations:** 1 Ocean and Earth Science, University of Southampton, National Oceanography Centre, Southampton, European Way, Southampton, SO14 3HZ UK; 2 Department of Zoology (Polychaete Group), Natural History Museum, London, SW7 5BD UK; 3 Monterey Bay Aquarium Research Institute, 7700 Sandholdt Road, Moss Landing, CA 95039 USA; 4 National Oceanography Centre, University of Southampton Waterfront Campus, European Way, Southampton, SO14 3HZ UK

## Abstract

Changes in habitat-forming organisms can have complex consequences for associated species. Sessile epibenthic glass “plate” sponges (Porifera: Hexactinellida) are conspicuous inhabitants of soft-sediment abyssal areas and their siliceous spicules create persistent spicule patches on the seafloor. Sponge spicule patch density, spatial dispersion, and percent cover were examined over a seven-year period (2006–2013) using remotely operated vehicle videos from Station M in the abyssal northeast Pacific (50˚00N, 123˚00W, ~4,000 m depth). There was an apparent large increase in newly dead plate sponges in February 2007 compared with December 2006, with this trend continuing through June 2007 (mean 0.03 % cover increasing to 0.33 %). A second increase in mean percent cover of dead plate sponges occurred from May 2011 (0.24 %) through June 2012 (0.60 %). Among the 28 megafaunal taxa occurring in association with the patches, the distributions of three taxa [two sponge taxa (Porifera) and brittle stars (Ophiuroidea)] suggested selectivity for the sponge spicule patches. The community structure of visible megafauna within sponge spicule patches was different when compared with that outside the patches suggesting that the sponges, after death, provide preferred habitat patches for certain benthic megafauna. These findings indicate that sponge spicule patches contribute to habitat heterogeneity in space and time.

**Electronic supplementary material:**

The online version of this article (doi:10.1007/s00227-014-2609-1) contains supplementary material, which is available to authorized users.

## Introduction

Characterizing deep-sea systems has become more important under changing climate and rising exploitation of deep-sea resources (Glover and Smith 2003; Thiel 2003; Ramirez-Llodra et al. 2011). Fine sediments dominate most deep seafloor habitats where sessile organisms often provide the only hard substrate available to other fauna and have the potential to create habitats (Beaulieu 2001b; Buhl-Mortensen et al. 2010). Such organisms are called ecosystem engineers (Jones et al. 1994; Wright and Jones 2006). Biological structures of ecosystem engineers increase habitat heterogeneity and complexity, thereby modifying species abundance and diversity (Levin and Dayton 2009) as exemplified by deep-sea coral reefs in canyons and continental margins (Buhl-Mortensen and Mortensen 2004). In sedimentary abyssal areas, however, such features are less obvious. Pennatulacea (sea pens), Xenophyophorea and Porifera (sponges) have been affirmed to be providers of habitats (Buhl-Mortensen et al. 2010). Some hexactinellid sponges (Porifera: Hexactinellida) also leave behind dense siliceous spicule patches, which themselves form persistent structures on the seafloor. To better understand processes shaping abyssal communities in space and time, we analyzed plate sponge spicule patch (SSP) density and size as well as megafaunal associations in a northeast Pacific abyssal area from 2006 to 2013.

One of the most studied abyssal areas is Station M (Sta. M) in the northeast Pacific, where long-term time series studies have been conducted since 1989. At Sta. M, changes in surface ocean conditions are translated to the deep ocean as particulate organic carbon (POC) flux variations (Smith et al. 2013), which have been correlated with megafauna community dynamics (Ruhl and Smith 2004; Kuhnz et al. 2014). In particular, large food influxes have been suggested as major community disturbances, increasing the density of a few species, while often decreasing the overall diversity (Kuhnz et al. 2014). Between 2006 and 2012, two major inputs of organic carbon occurred, followed by a community shift from a sessile, suspension feeding, sponge-dominated community to a mobile, detritus-feeding, holothurian-dominated community. By late 2011, the densities of mobile organisms had increased by nearly an order of magnitude, while diversity was well below 2006 levels (Kuhnz et al. 2014). Less is known about the temporal dynamics of sessile megafauna that build biogenic hard structures.

**Fig. 1:**
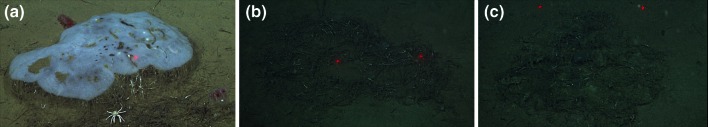
Monterey Bay Aquarium Research Institute (MBARI) video annotation and reference system high-definition framegrabs **a** living plate sponge *Bathydorus laniger* **b** sponge spicule patch **c** sponge spicule patch with tissue remains

So far, the most studied deep-sea sponge group is the Hexactinellida (glass) plate sponges (Kahn et al. 2012): *Bathydorus laniger* (Hexactinellida: Rossellidae, Fig. 1a) and *Docosaccus* *maculatus* (Hexactinellida: Euplectellidae). Both sponges have very similar gross morphology: a plate-like form with basalia, which stilt the body a few centimeters above the seafloor (Kahn et al. 2013). Using towed camera-sled images, Kahn et al. (2012) found that both of these plate sponge species displayed inter-annual variations in density and average body size between 1989 and 2005. Density changes were correlated with POC flux 13 months earlier and with the North Oscillation Index (NOI, an ENSO indicator) 15 months earlier prior to the changes in sponge density. Although not yet well quantified, living plate sponges have the potential to create habitats, suggesting that if plate sponge density varies over time, it might impact other organisms (Wulff 2006; Buhl-Mortensen et al. 2010).

Videos acquired by remotely operated vehicles (ROVs) since 2006 and sediment push cores from Sta. M have shown that the remains of these dead plate sponges form sponge spicule patches (SSPs) on the seafloor (Fig. 1b). Sponge spicules are resistant to dissolution (Kamatani 1971; Maldonado et al. 2005). Bett and Rice (1992) reported SSPs from *Pheronema carpenteri* (Hexactinellida: Pheronematidae) covering up to one-third of the sediment surface in the Porcupine Seabight in the northeast Atlantic. Here, the faunal community within the patches was substantially modified and occurred in greater densities compared with the background macrobenthos. At Sta. M, Beaulieu (2001a) analyzed another hexactinellid sponge, *Hyalonema bianchoratum*, characterized by a long stalk holding not only the sponge body several centimeters above the seafloor, but also an assemblage of suspension feeders. Overall, 144 taxa were associated with *H. bianchoratum* stalks, with an average of 4.1 taxa per stalk, which were dominated by Foraminifera, polychaete worms, peracarid shrimp and mollusks (Beaulieu 2001a).

Given the potential importance of deep-sea sponges as habitats and their links to environmental forcing, some key questions arise. This study examines temporal variations in SSP density, percent cover and size at Sta. M from 2006 to 2013. Using ROV videos recorded over 7 years, two main questions were addressed: (1) Did SSP change in terms of density and percent cover over time? (2) Were any megafauna associated obligatorily or preferentially with SSPs and if so, did the association change over time?

## Materials and methods

### Video acquisition

From December 2006 to June 2013, ten research cruises were conducted at Sta. M (50˚00 N, 123˚00 W, ~4,000 m depth, Table 1, Kuhnz et al. 2014) in the northeast Pacific. Overall, silty clay particles dominated sediments and little topographic relief was found over large areas (<60 m relief over 770 km^2^, Smith et al. 1993). Seabed video footage was acquired using the Monterey Bay Aquarium Research Institute (MBARI) ROV *Tiburon* (December 2006–September 2007) and ROV *Doc Ricketts* (February 2009–June 2013). A total of 16 transects (>16,500 m^2^) were recorded using Ikegama high-definition cameras fitted with HA10Xt.2 Fujinon lenses and lasers spaced 29 cm apart to assess the field of view. Transect lengths varied from 80 to 4,500 m, while the transect width was kept close to 1 m using the ROV lasers as a guide.

**Table 1:** Sampling details for quantitative ROV transects at Sta. M: year, month, ROV dive number with T for ROV *Tiburon* and D ROV *Doc Ricketts*, transect area and #Bins as number of 20 m^2^ subsamples

Year	Month	Dive number	Combined transect area (m^2^)	#Bins
2006	December	T1067	1,120	56
2007	February	T1077, T1080	220	11
	June	T1094	80	4
	September	T1141, T1143	420	21
2009	February	D008	1,560	1
2011	May	D230, D232	4,500	225
	November	D321, D323, D324	2,640	132
2012	June	D403	400	20
	November	D442, D443	2,640	130
2013	June	D486	3,000	150

Videos were analyzed using MBARI video annotation and reference system (VARS) software (Schlining and Stout 2006), which allowed us to manually annotate videos and store notes directly in a database. Each SSP was noted along with its geographic location, position within the transect and depth. Biogenic structures such as mounds or plate sponge remains with tissue (Fig. 1c) were also noted. Only SSPs and organisms that passed the lasers and visible in the lower 75 % of video were taken into account to ensure a consistent meter-wide strip in the oblique view (Kuhnz et al. 2014). Patch annotations were conservative in that only obvious patches with a clear aggregation of sponge spicules were used for data analysis. The density of spicules within each patch could not be objectively counted from the video.

### Density data and spatial dispersion

SSP density was defined as the mean number of patches m^−2^. To obtain the patch mean density and associated standard error (SE) for each time period, each transect was divided into contiguous 20-m^2^ bins (pseudoreplicates of 20 m of transect length by 1 m transect width). The bins were chosen in accordance with the ROV navigation data resolution as used in a recent megafauna study (Kuhnz et al. 2014). Abundances within bins were analyzed as replicate sample units to calculate mean densities. Temporal differences in density were assessed using a Kruskal–Wallis test (*H*, *P*, SigmaPlot version 12.5, Sokal and Rohlf 1995) as the data did not meet normality criteria. Correlations between SSP densities were also tested with living plate sponge densities during the same time period (*r*s, *N*, *P*, Spearman correlation, SigmaPlot version 12.5, Sokal and Rohlf 1995). The coefficient of dispersion (CD, variance/mean ratio) of individual patches was determined for each sampling period. By comparing the coefficient of dispersion of patches to a random distribution (Poisson distribution, CD = 1), the evenness of patch spatial distribution was assessed (Elliott 1971).

### Percent cover data

Patch sizes were measured from framegrabs taken with VARS. For each SSP, a framegrab was taken with the ROV lasers centered at mid-length of the patch. Patch shape was approximated as a rectangular area and estimated based on the distance of the lasers in the image (Online resource 1). With this method, overall repeatability and horizontal measurements were near 100 % accuracy, while vertical measurements could be less accurate (1–5 %) due to the oblique view of ROV video (Wakefield and Genin 1987). To ensure less distortion for large patches or for patches larger than the camera frame, several video frames were stitched together to obtain total patch area.

SSP mean percent cover over time was calculated as the percentage of area covered by all patches for a transect area. Temporal changes were assessed using a Kruskal–Wallis test. The relationship between patch density and patch size was investigated. The influence of biogenic features (mounds and sponge tissue remains) on patch size was investigated using a Mann–Whitney *U* test (*U*, *N1*, *N2*, *P*, SigmaPlot version 12.5, Sokal and Rohlf 1995). The size distributions of SSPs were computed and compared over time by computing the distance between the curves (DOMDIS routine, Primer version 6, Clarke 1990; Clarke and Gorley 2006) and conducting a multivariate analysis of similarity (ANOSIM, *R*, *P*, Primer version 6, Clarke 1993; 999 permutations employed).

### Megafaunal associations

Living megafaunal organisms observed within and outside each patch were identified to the lowest possible taxon. To ensure the best and most consistent detection over sampling periods, only high-definition videos were used, thereby excluding the February 2007 transect for which only standard definition video was available. To determine which organisms might show habitat selection within spicule patches, the densities of the specific organisms living within and outside the patches were tested for differences (Mann–Whitney *U* test). Data outside patches were only available until November 2012. The densities of specimens outside the patch were deduced from the total density of organisms recorded for each transect (density corrected for the transect area minus total patch area). Taxa with a significantly lower open space density were considered as significant associates.

The structures of communities inside and outside of patches were analyzed using multivariate ordination techniques (Primer version 6). Compositions as Bray–Curtis similarity of the square root-transformed density data inside and outside patches were computed over time. A group average cluster analysis was then applied to each similarity matrix. The correlation between the two community structures over time was assessed using a Mantel test (Relate routine, *ρ*, *P*, Primer version 6). We also investigated whether SSPs with mounds or sponge remains had a different density of significant associates than SSPs without biogenic features (Mann–Whitney *U* test).

## Results

### Patch observations

SSPs appeared like islands projecting one or more centimeters above the surrounding sediment. It was not possible to identify which species of plate sponge created individual spicule patches. The density of the glassy spicules appeared to vary but could not be quantified from video. Overall, we observed 101 SSPs during the time series (Fig. 1b). No clustering of patches was found (patches were generally several meter or more apart), as indicated by the coefficient of dispersion (0.38–0.90). The average patch area was 0.27 m^2^ (0.19–0.48 m^2^) and did not change significantly over time. Two main biogenic structures were frequently seen within patches. Mounds created by other organisms occurred beneath 18 % of the patches with the highest frequency observed in June 2007 when 50 % of patches had mounds. The remains of plate sponge tissues were visible in 30 % of patches over the time series (Fig. 1c) with a maximum of 63 % in December 2006. No sponge tissue remains were observed within patches in February 2007.

### Density and percent cover

**Fig. 2:**
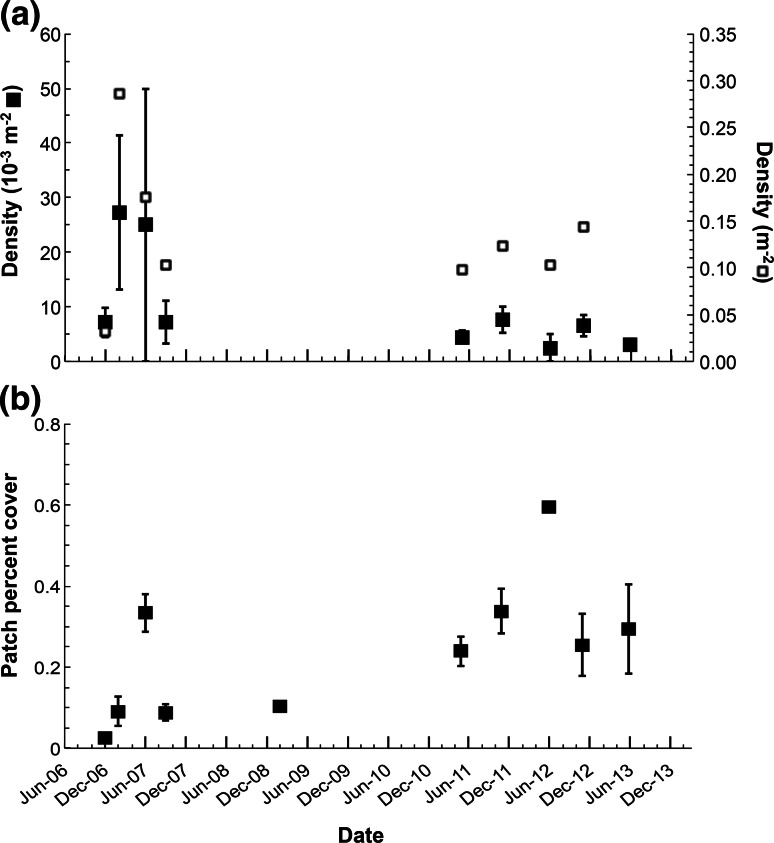
Sponge spicule patch features in time. **a** mean density as 10^−3^ number of patch m^−2^ with SE in *black squares* compared with living plate sponge density in *open squares*, **b** mean percent cover of sponge spicule patches within transects with SE. Horizontal axis with 6-month major interval tick and 2-month minor interval ticks. At each date, *n* = #Bins of date, see Table 1. No living plate sponge data available for June 2013

SSP density differed significantly among sampling periods (Fig. 2a). The highest density was found in February 2007 with 27 ± 14 × 10^−3^ patches m^−2^, *N* = 11 and the lowest in June 2012 with 2.5 ± 2 × 10^−3^ patches m^−2^, *N* = 20. The decrease in patch density over time was statistically significant (*H* = 17.606, *P* = 0.024). There was no significant correlation between SSPs and living plate sponge densities (*r*s < 0.1, *N* = 9, *P* > 0.05).

The amount of seafloor covered in SSPs changed over time (Fig. 2b). The lowest percent cover was in December 2006 when patches covered a mean of 0.02 ± 0.009 % of the transect area, *N* = 56 bins. The highest percent cover was observed in June 2011 at 0.59 ± 0.39 %, *N* = 225 bins. The temporal variations in patch spatial coverage were significant (*H* = 36.185, *P* < 0.001) and the differences were driven by the 2007 observations.

SSP density and mean size were not significantly correlated (*r*s = −0.259, *N* = 9, *P* = 0.462). The patch size frequency distributions for each time period were not found to be significantly different (*R* = 0.173, *P* = 0.319). The most even size distribution was in September 2007, whereas in June 2007, 2012, 2013 and November 2012, the distributions tended to be dominated by one size class. Except in June 2012, the dominant size classes were the smallest: 0–0.17 and 0.18–0.35 m^2^. No difference was found between the mean size of a patch when mounds were present (*U* = 544, *N1* = 18, *N2* = 81, *P* = 0.094). Patches with plate sponge tissue remains were significantly larger than those without tissue (*U* = 684, *N1* = 29, *N2* = 70, *P* = 0.011) with a median of 0.229 versus 0.154 m^2^.

### Megafaunal associations

**Fig. 3:**
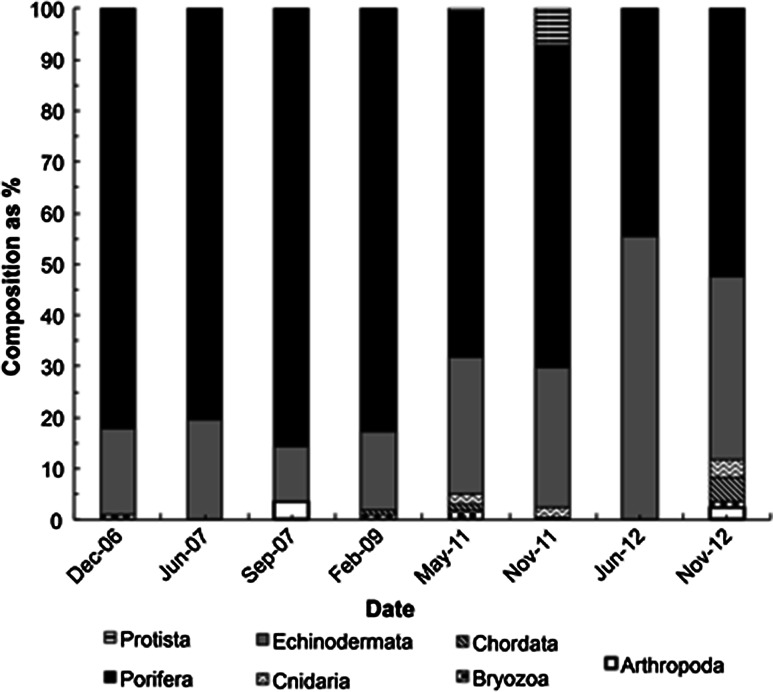
Sponge spicule patch assemblage composition by phylum over time as percentage. Arthropoda in *white*, Bryozoa in *black* and *white squares*, Chordata in *tilted black lines*, Cnidaria in *black chevrons*, Echinodermata in *gray*, Porifera in *black*, Protista in *black horizontal* *lines*

**Fig. 4:**
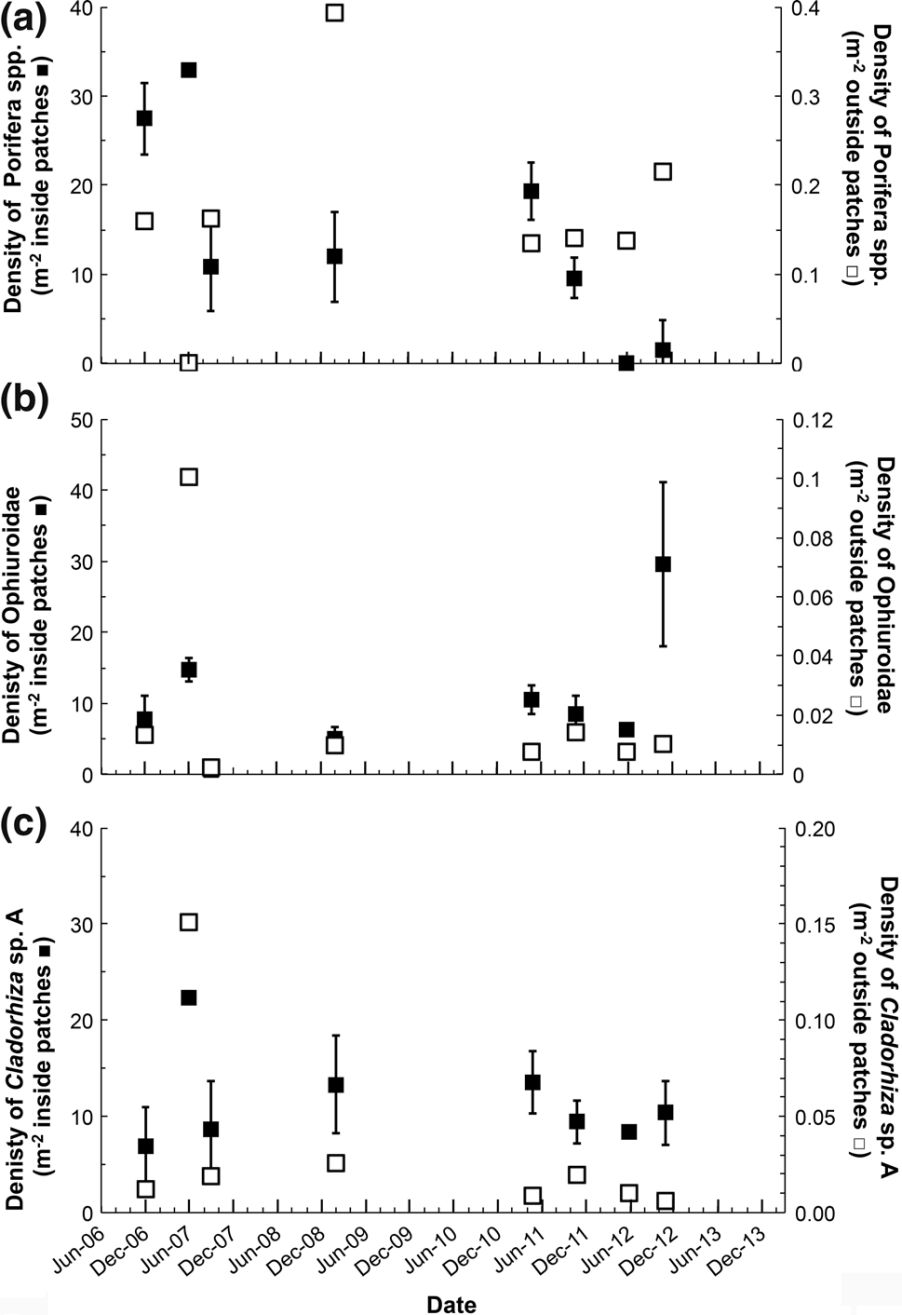
Statistically significant megafauna associates of sponge spicule patch: mean densities per m^−2^ of patch (*filled square*; *left axes*) and m^−2^ outside of patch (*open square*; *right axes*) in time with SE **a** Porifera spp. **b** *Cladorhiza* sp. A **c** Ophiuroidea. Horizontal axis with 6-month major interval tick and 2-month minor interval ticks. At each date, *n* = #Bins of date, see Table 1

Overall, 28 taxa were observed within the patches (Online resource 2) mainly Porifera, 72 % (44–86 %) and Echinodermata, 24 % (11–55 %). These taxa constitute only a subset of the megafauna known to occur at Station M. The relative dominance of Porifera decreased over time (Fig. 3). Among these invertebrates, three taxa had densities significantly higher within patches than outside patches, i.e., they were significant associates (Fig. 4). First, the strongest association was found for the group Porifera spp. (*U* = 7.5, *N1* = 8, *N2* = 8, *P* = 0.007) with medians of 10.300 ind. m^−2^ in patches versus 0.150 ind. m^−2^ out of patches. This group was composed of small bulbous Porifera not readily identifiable to species. The highest density of these sponges within patches was found in November 2012 with 29 ± 11 × 10^−3^ ind. m^−2^, *N* = 130; the lowest density was recorded in September 2007 with 1 ± 1 × 10^−3^ ind. m^−2^, *N* = 21 (Fig. 4a). These Porifera spp. occurred in greater abundance in patches with dead sponge tissue remains compared with those without tissue remains (*U* = 655, *N1* = 28, *N2* = 63, *P* = 0.044). Second, juveniles of the sponge *Cladorhiza* sp. A were also found in higher density within patches than outside of patches (*U* = 9, *N1* = 8, *N2* = 8, *P* = 0.015) with medians of 0.894 ind. m^−2^ within patches versus 0.019 ind. m^−2^ outside patches (Fig. 4b). Within patches, the highest density of *Cladorhiza* sp. A occurred in June 2007 with 22 ± 1 × 10^−3^ ind. m^−2^, *N* = 4, whereas the lowest density was found in December 2006 with 7 ± 4 × 10^−3^ ind. m^−2^, *N* = 56. Lastly, Ophiuroidea densities were higher within patches (*U* = 9, *N1* = 8, *N2* = 8, *P* = 0.015) with a median 0.609 ind. m^−2^ inside patches versus 0.012 ind. m^−2^ outside of patches (Fig. 4c). Ophiuroidea density within patches varied from 0 ind. m^−2^ (*N* = 20, June 2012) to 33 ± 33 ind. m^−2^ (*N* = 4, June 2007).

None of the significantly associated taxa had different densities in patches with and without mounds (*P* > 0.05). Overall, the community structures of the associates inside and outside patches were different over time (Relate test, *ρ* = 0.382, *P* = 0.062).

## Discussion

The density and percent cover of plate sponge spicule patches on the seafloor at Station M varied significantly over the study period, but patch size did not. This reflected changes in living plate sponge populations. Both SSP densities and areas were of the same order of magnitude as the live sponges observed by Kahn et al. (2012). Although the degradation or dispersal of spicules has not been studied in these species, individual SSP size is thought to decrease over time from an initial maximum established by the plate sponge at time of death. The correlation between SSP density and mean size was not significant in this study, however, it is worth noting that the smallest SSPs occurred in February 2007 when SSP density was the highest and the largest in June 2012 when SSP density was the lowest. This pattern might relate to the negative correlation between living plate sponge density and size reported by Kahn et al. (2012).

As previously reported for living sponges (Kahn et al. 2012), this study shows that SSPs are randomly dispersed on the seafloor with no obvious living plate sponges within them. The random distribution suggests that the soft-sediment habitat at Sta. M was homogeneous in terms of the settlement and/or the survival of plate sponges. This was also found with *Hyalonema bianchoratum* sponges living there (Beaulieu 2001a). Other factors such as potentially different timescales of living sponge settlement and growth versus decay might also explain the lack of correlation between living plate sponge and SSP densities. Overall, between 1989 and 2005, no correlation was found between living plate sponge and SSP densities by Kahn et al. (2012) although higher density of SSPs occurred in years with higher living sponge density.

SSP epibenthic megafaunal assemblages were composed of 28 taxa, three of which were classified as significant associates. There was no obligate association with patches as was seen for living sponge macrofaunal and megafaunal assemblages in other systems (Wulff 2006; Buhl-Mortensen et al. 2010; Gutt et al. 2013). Obligate epibionts are generally rare (Wahl and Mark 1999), but video data are limited by the difficulties in discerning some taxa in photos and assigning a morphotype to taxa with few individuals. Species that are small, are camouflaged or have a burrowing life style are likely underrepresented in the data. SSP sampling will be required to confirm the identification of the epibenthic megafaunal taxa seen on videos, including taxa not readily observable in pictures.

SSPs may benefit this abyssal community by expanding the realized niche of several species otherwise living in sub-optimal conditions. Bett and Rice (1992) suggested that SSPs in the Porcupine Seabight had three main effects on other fauna: providing refuge, higher food supply and substrate. Our finding that many ophiuroids were living with their central disks protected within the spicules and their filter-feeding arms emerging into the water has also been reported in shallower systems (Haanes and Gulliksen 2011). Associated fauna might also avoid predation because of the protection offered by the spicule canopy (Wulff 2006). In addition, Ophiuroidea associate with deep-sea hard structures thereby gaining access to higher food flux and enhanced filter feeding (Levin 1991; Buhl-Mortensen et al. 2010). The spicule network might also retain more phytodetritus than surrounding sediment benefiting surface and subsurface deposit feeders (Bett and Rice 1992). In Antarctica, Gutt et al. (2013) reported a significant positive correlation between the number of epi-macrofauna taxa and spicule percent cover, where the epi-macrofauna had a higher re-colonization rate in spicule patches than megafauna. This enriched food environment might provide small preys for the carnivorous *Cladorhiza* sp. A (Vacelet 2007).

Differential settling of associated fauna may also occur, with SSPs providing an advantageous substrate for other sponges to settle (Barthel 1992; Leys et al. 2004). Some sponges, in particular demosponge species (Porifera: Demospongiae), are unable to colonize open sediments (Barthel 1992). *Cladorhiza* sp. A specimens observed within patches were mainly small and possibly juveniles. And the Porifera spp. group observed in SSPs could be either small individuals of other known sponges at the site (such as *Bathydorus laevis spinosu*s, *Euplectella* spp.) or of new small species (Kuhnz et al. 2014). In Antarctica, the abundance of larger Hexactinellida decreased with spicule cover, whereas small sponge density increased with spicule cover (Gutt et al. 2013). In addition, SSPs might locally increase dissolved silicon (Si) although hexactinellid spicules can have low dissolution rates (Hurd 1973; Chu et al. 2011). Many sponges have been reported to be strongly limited by Si ambient availability (Maldonado et al. 2005, 2012) and high dissolved silicate is an important factor for juvenile settlement (Leys et al. 2004, 2007).

Overall, SSPs created small-scale habitats, typically <1 m^2^. The community structures of the complete assemblage (28 taxa) and of the significant associates changed over time inside and outside spicule patches, enhancing spatial heterogeneity. We observed that SSPs appeared to be at different stages of decay and colonization, providing temporal heterogeneity. Spatiotemporal heterogeneity and colonization are known to increase diversity as proposed in the patch mosaic theory, which suggests that seafloor small-scale disturbances permit high local diversity by creating successional sequences that are temporally out of phase (Grassle and Sanders 1973; Grassle and Morse-Porteous 1987; Rex and Etter 2010).

In conclusion, SSPs at Sta. M varied over the seven-year study period in terms of density and percent cover, while their size did not. Notably, the number of SSPs decreased between December 2006 and June 2013, whereas their percent cover increased over that period. These changes may follow climate-driven changes in food supply known to have occurred over the same period. SSPs provide microhabitats on the abyssal seafloor, locally influencing megafaunal density and impacting Sta. M ecology. Further sampling will be needed to fully characterize the megafaunal species composition observed within SSPs and to determine whether sponge spicule density increases patch diversity and the importance of SSPs to smaller fauna.

## Electronic supplementary material

Below is the link to the electronic supplementary material.

Supplementary material 1 (PDF 61 kb)

Supplementary material 2 (PDF 89 kb)

## References

[CR1] Barthel D (1992). Do hexactinellids structure Antarctic sponge associations?. Ophelia.

[CR2] Beaulieu SE (2001). Colonization of habitat islands in the deep sea: recruitment to glass sponge stalks. Deep Sea Res I.

[CR3] Beaulieu SE (2001). Life on glass houses: sponge stalk communities in the deep sea. Mar Biol.

[CR4] Bett BJ, Rice AL (1992). The influence of hexactinellid sponge (*Pheronema carpenteri*) spicules on the patchy distribution of macrobenthos in the Porcupine Seabight (bathyal NE Atlantic). Ophelia.

[CR5] Buhl-Mortensen L, Mortensen PB (2004). Crustaceans associated with the deep-water gorgonian corals *Paragorgia arborea* (L., 1758) and *Primnoa resedaeformis* (Gunn., 1763). J Nat Hist.

[CR6] Buhl-Mortensen L, Vanreusel A, Gooday AJ, Levin LA, Priede IG, Buhl-Mortensen P, Gheerardyn H, King NJ, Raes M (2010). Biological structures as a source of habitat heterogeneity and biodiversity on the deep ocean margins. Mar Ecol.

[CR7] Chu JWF, Maldonado M, Yahel G, Leys SP (2011). Glass sponge reefs as a silicon sink. Mar Ecol Prog Ser.

[CR8] Clarke KR (1990). Comparisons of dominance curves. J Exp Mar Biol Ecol.

[CR9] Clarke KR (1993). Non-parametric multivariate analyses of changes in community structure. Aust J Ecol.

[CR10] Clarke KR, Gorley RN (2006). Primer v6: user manual/tutorial.

[CR11] Elliott JM (1971) Some methods for the statistical analysis of samples of benthic invertebrates. Freshwater Biological Association. Scientific publication 25, Ambleside, UK

[CR12] Glover AG, Smith CR (2003). The deep-sea floor ecosystem: current status and prospects of anthropogenic change by the year 2025. Environ Conserv.

[CR13] Grassle JF, Morse-Porteous LS (1987). Macrofaunal colonization of disturbed deep-sea environments and the structure of deep-sea benthic communities. Deep Sea Res A.

[CR14] Grassle JF, Sanders HL (1973). Life histories and the role of disturbance. Deep Sea Res Oceanogr Abstr.

[CR15] Gutt J, Böhmer A, Dimmler W (2013). Antarctic sponge spicule mats shape macrobenthic diversity and act as a silicon trap. Mar Ecol Prog Ser.

[CR16] Haanes H, Gulliksen B (2011). A high local species richness and biodiversity within high-latitude calcareous aggregates of tube-building polychaetes. Biodivers Conserv.

[CR17] Hurd DC (1973). Interactions of biogenic opal, sediment and seawater in the Central Equatorial Pacific. Geochim Cosmochim Ac.

[CR18] Jones CG, Lawton JH, Shachak M (1994). Organisms as ecosystem engineers. Oikos.

[CR19] Kahn AS, Ruhl HA, Smith KL (2012). Temporal changes in deep-sea sponge populations are correlated to changes in surface climate and food supply. Deep Sea Res I.

[CR20] Kahn AS, Geller JB, Reiswig HM, Smith KL (2013). *Bathydorus laniger* and *Docosaccus maculatus* (Lyssacinosida; Hexactinellida): two new species of glass sponge from the abyssal eastern North Pacific Ocean. Zootaxa.

[CR21] Kamatani A (1971). Physical and chemical characteristics of biogenous silica. Mar Biol.

[CR22] Kuhnz LA, Ruhl HA, Huffard CL, Smith KL (2014). Rapid changes and long-term cycles in the benthic megafaunal community observed over 24 years in the abyssal northeast Pacific. Progr Oceanogr.

[CR23] Levin LA (1991). Interactions between metazoans and large, agglutinating protozoans: implications for the community structure of deep-sea benthos. Am Zool.

[CR24] Levin LA, Dayton PK (2009). Ecological theory and continental margins: where shallow meets deep. Trends Ecol Evol.

[CR25] Leys SP, Wilson K, Holeton C, Reiswig HM, Austin WC, Tunnicliffe V (2004). Patterns of glass sponge (Porifera, Hexactinellida) distribution in coastal waters of British Columbia, Canada. Mar Ecol Prog Ser.

[CR26] Leys SP, Mackie GO, Reiswig HM (2007). The biology of glass sponges. Adv Mar Biol.

[CR27] Maldonado M, Carmona MC, Velásquez Z, Puig A, Cruzado A, Lόpez A, Young CM (2005). Siliceous sponges as a silicon sink: an overlooked aspect of benthopelagic coupling in the marine silicon. Limnol Oceanogr.

[CR28] Maldonado M, Ribes M, van Duyl FC (2012). Nutrient fluxes through sponges: biology, budgets, and ecological implications. Adv Mar Biol.

[CR29] Ramirez-Llodra E, Tyler PA, Baker MC, Bergstad OA, Clark MR, Escobar E, Levin LA, Menot L, Rowden AA, Smith CR, Van Dover CL (2011). Man and the last great wilderness: human impact on the deep sea. PLoS One.

[CR30] Rex MA, Etter RJ (2010). Deep-sea biodiversity: pattern and scale.

[CR31] Ruhl HA, Smith KL (2004). Shifts in deep-sea community structure linked to climate and food supply. Science.

[CR32] Schlining BM, Stout NJ (2006) MBARI’s video annotation and reference system. In Oceans 2006 IEEE pp 1–5

[CR33] Smith KL, Kaufmann RS, Wakefield WW (1993). Mobile megafaunal activity monitored with a time-lapse camera in the abyssal North Pacific. Deep Sea Res I.

[CR34] Smith KL, Ruhl HA, Kahru M, Huffard CL, Sherman AD (2013). Deep ocean communities impacted by changing climate over 24 y in the abyssal northeast Pacific Ocean. Proc Natl Acad Sci USA.

[CR35] Sokal RR, Rohlf FJ (1995). Biometry.

[CR36] Thiel H, Tyler PA (2003). Anthropogenic impacts on the deep sea. Ecosystems of the deep oceans. Ecosystems of the world 28.

[CR37] Vacelet J (2007) Diversity and evolution of deep-sea carnivorous sponges. In: Custódio MR, Lôbo-Hajdu G, Hajdu E, Muricy G (eds) Porifera research: biodiversity, innovation and sustainability, Série Livros 28:107–115

[CR38] Wahl M, Mark O (1999). The predominantly facultative nature of epibiosis: experimental and observational evidence. Mar Ecol Progr Ser.

[CR39] Wakefield WW, Genin A (1987). The use of a Canadian (perspective) grid in deep-sea photography. Deep Sea Res A.

[CR40] Wright JP, Jones CG (2006). The concept of organisms as ecosystem engineers ten years on: progress, limitations, and challenges. Bioscience.

[CR41] Wulff JL (2006). Ecological interactions of marine sponges. Can J Zool.

