# Supplementary material for: Spatial and temporal variation in sponge spicule patches at Station M, northeast Pacific
Source: Mar Biol. 2015 Jan 22;162(3):617–24. doi: 10.1007/s00227-014-2609-1 (PMC4325134; doi:10.1007/s00227-014-2609-1)
Supplement: Supplementary file 1 — Supplementary material 1 (PDF 61 kb) [file 227_2014_2609_MOESM1_ESM.pdf]

Spatial and temporal variation in sponge spicule patches at Station M, northeast Pacific. *Marine Biology*. Laguionie-Marchais, C.*, Kuhnz, L.A., Huffard, C.L., Ruhl, H.A., Smith, K.L.Jr.

*Corresponding author: University of Southampton, National Oceanography Centre, Southampton, European Way, Southampton, SO14 3HZ, UK & Natural History Museum, Department of Zoology (Polychaete Group), London SW7 5BD, UK; Claire.Laguionie-Marchais@noc.soton.ac.uk

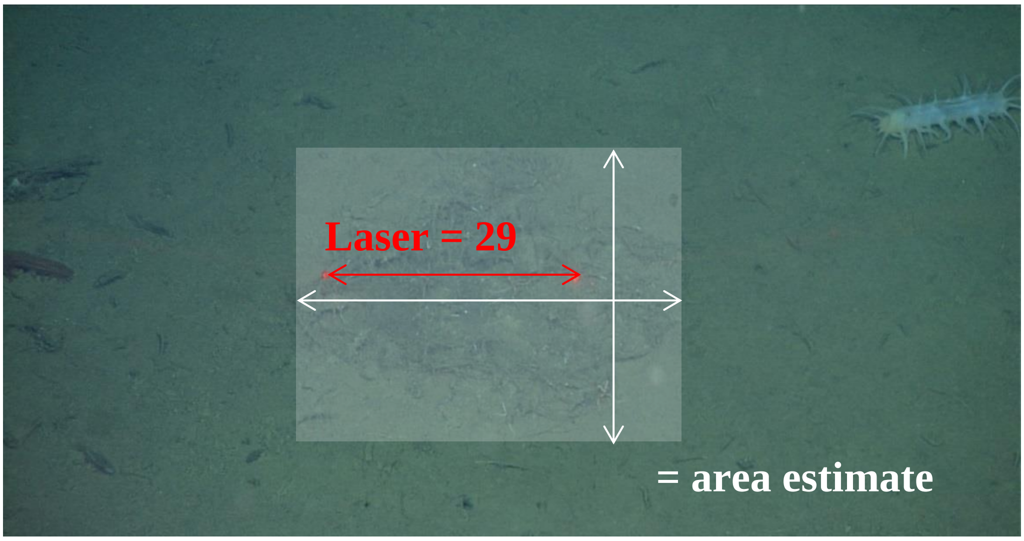

Online ressource 1 Method for measuring sponge spicule patch sizes using Monterey Bay Aquarium Research Institute video annotation and reference system. ROV laser pointers (red dots) aligned with patch center to minimize distortion. Patch area estimate as white rectangle area
