# Supplementary material for: Spatial and temporal variation in sponge spicule patches at Station M, northeast Pacific
Source: Mar Biol. 2015 Jan 22;162(3):617–24. doi: 10.1007/s00227-014-2609-1 (PMC4325134; doi:10.1007/s00227-014-2609-1)
Supplement: Supplementary file 2 — Supplementary material 2 (PDF 89 kb) [file 227_2014_2609_MOESM2_ESM.pdf]

Spatial and temporal variation in sponge spicule patches at Station M, northeast Pacific. *Marine Biology*. Laguionie-Marchais, C.[*], Kuhnz, L.A., Huffard, C.L., Ruhl, H.A., Smith, K.L.Jr.

*Corresponding author: University of Southampton, National Oceanography Centre, Southampton, European Way, Southampton, SO14 3HZ, UK & Natural History Museum, Department of Zoology (Polychaete Group), London SW7 5BD, UK; Claire.Laguionie-Marchais@noc.soton.ac.uk

Online resource 2 List of organisms inhabiting sponge spicule patches observed in ROV video between December 2006–November 2012. Presumptive organisms as one morpho-species unless otherwise denoted by spp. and standardized with Kuhnz et al. (2014). All organisms identified to lowest possible taxonomic category. Sponge spicule patch megafaunal significant associates in bold.

Major Group	Group	Taxon
Foraminifera	Xenophyophoroidea	Psamminidae spp.
Porifera	Porifera	**Porifera spp.**
	Hexactinellida	*Bathydorus laniger*
		Bathydorus spp.
		Docosaccus maculatus
		Euplectellidae spp.
		Hyalonema bianchoratum
	Demospongiae	***Cladorhiza* sp. A**
Cnidaria	Actiniaria	*Bathyphellia australis*
	Anthomedusae	Hydractiniidae sp.
	Zoanthidea	*Epizoanthus stellari*
Echiura	Echiura	Echiura sp.
Arthropoda	Decapoda	*Munidopsis* sp.
	Isopoda	Isopoda sp.
		Munnopsidae sp.
	Pycnogonida	Pycnogonida sp.
Bryozoa	Bryozoa	*Striatodoma dorothea*
Echinodermata	Asteroidea	Asteroidea sp.
	Crinoidea	Bathycrinidae sp.
		Fariometra parvula
	Echinoidea	*Cystechinus loveni*
		Cystocrepis setigera
	Holothuroidea	*Abyssocucumis abyssorum*
		Elpidia sp. A. (new species)
		Peniagone sp. A (new species)
	Ophiuroidea	**Ophiuroidea spp.**
Chordata	Tunicata	*Culeolus* sp.
	Ascidiacea	Pyuridae sp.

Kuhnz LA, Ruhl HA, Huffard CL, Smith KL Jr (2014) Rapid changes and long-term cycles in the benthic megafaunal community observed over 24 years in the abyssal northeast Pacific. Progr Oceanogr 124:1–11. doi:10.1016/j.pocean.2014.04.007
